# A commentary on ‘Core decompression combined with platelet-rich plasma-augmented bone grafting for femur head necrosis: a systematic review and meta-analysis’

**DOI:** 10.1097/JS9.0000000000001437

**Published:** 2024-04-04

**Authors:** Zhe Shen, Yawen He, Lianguo Wu

**Affiliations:** a The Second Clinical Medical College of Zhejiang Chinese Medical University; b Hangzhou Clinical Medical College of Zhejiang Chinese Medical University; c Department of Orthopaedics, The Second Affiliated Hospital of Zhejiang Chinese Medical University, Hangzhou, Zhejiang, People’s Republic of China

*Dear Editor,*

We are interested in the article entitled ʻCore decompression combined with platelet-rich plasma-augmented bone grafting for femur head necrosis: a systematic review and meta-analysisʼ, which was published in the *International Journal of Surgery* ^1^. The meta-analysis included 11 randomized controlled trials (RCT) comparing core decompression (CD) and bone grafting combined with or without platelet-rich plasma (PRP). Their findings suggest that the combination of core decompression and bone grafting with PRP is superior to the approach without PRP, demonstrating enhanced effectiveness in terms of function, pain relief, and radiographic progression. Additionally, it results in lower rates of treatment failure and adverse events. These findings are both timely and of great significance. While the rigorous efforts and valuable contributions of this study are deeply appreciated, this article has raised some concerns for us.

Firstly, most of the randomized controlled trials included in this article were followed up for only 6 months to 12 months (8 of 11). A recent retrospective study^2^ with a follow-up period of 60 months showed that significant results were noted in favor of PRP treatment compared with controls based on the mean VAS (visual analog scale) scores at the 6 months (2.9±0.7 vs. 1.9±0.6, *P*<0.01) and final follow-up postoperative (2.8±1.2 vs. 2.2±0.7, *P*=0.04). The mean HHS (Harris hip score) in group A was significantly lower than controls at the 6 months (80.5±13.8 vs. 89.8±12.8, *P*=0.02). However, at the final follow-up, there is no significant difference between the groups (77.0±12.4 vs. 83.1±9.3, *P*=0.07). Therefore, it is hoped that there will be more long-follow-up randomized controlled trials to evaluate the efficacy of CD and bone grafting combined with PRP in the future.

Secondly, in the ‘Methods’ section, the authors said that they searched seven databases for relevant studies published until September 2022. However, the paper was accepted on 11 December 2023, so it seems that the search was stopped prematurely, considering the date of publication. Thus, there should be an updated list of articles retrieved from the databases before the article is accepted for publication.

Finally, the screening process seems flawed, as the sum of included and excluded studies doesn’t equal the initial records.

We thank Zhu *et al*. again for their meaningful work in summarizing the evidence of the combination of core decompression and bone grafting with PRP in the treatment of the femoral head. Our suggestions are merely to further refine an already outstanding piece of research. We eagerly await more innovative works from the authors in the future.

## Ethical approval

Not applicable.

## Consent

Not applicable.

## Sources of funding

Not applicable.

## Author contribution

Z.S., Y.H., and L.W.: conception and design of the study; Z.S. and Y.H.: collected and analyzed the data; Z.S. and Y.H.: wrote the paper. All the authors were involved in drafting and revising the paper and finally read and approved the final manuscript.

## Conflicts of interest disclosure

There are no conflicts of interest.

## Research registration unique identifying number (UIN)

Not applicable.

## Guarantor

Lianguo Wu accepts full responsibility for the work and the conduct of the study.

## Data availability statement

This manuscript is a comment. Don’t need a Data availability statement. However, all the data from the current study are publicly available.

## Provenance and peer review

Not applicable.
